# Experimental study on soil impermeability based on chemical improvement method

**DOI:** 10.1371/journal.pone.0334100

**Published:** 2025-10-21

**Authors:** Hai Sang, Bin Xiong, Chuan Jin, Deying Tang, Neng Xiong, Yifan Chen

**Affiliations:** 1 China Communications Construction First Highway Engineering Co., Ltd., Zhengzhou, Henan, China; 2 College of River and Ocean, Chongqing Jiaotong University, Chongqing, China; Ardakan University, IRAN, ISLAMIC REPUBLIC OF

## Abstract

To address the challenge of high permeability in coastal silty sand foundation pits, which is prone to seepage failure, this study investigated the improvement effects of three chemical stabilizers—sodium silicate, calcium lignosulfonate, and sodium polyacrylate—on the permeability characteristics of undisturbed silty sand using a self-designed seepage test system. The results demonstrated that the cumulative erosion mass of the untreated soil increased exponentially over time, with an erosion rate as high as 89.39 g/min. In contrast, the cumulative erosion mass of the silty sand treated with any of the three chemical stabilizers exhibited a trend of initial increase followed by stabilization, and the erosion rates were all reduced to below 30 g/min. Notably, under the conditions of 4% dosage and 6 hours of curing, sodium polyacrylate reduced the erosion rate to 3.98 g/min, limited the cumulative erosion mass to only 35 g, stabilized the permeability coefficient within the range of 10 ⁻ ⁶ to 10 ⁻ ⁷ cm/s, and shortened the permeability stabilization time to 6.3 minutes. Its improvement effect was superior to that of the other two stabilizers, meeting the anti-seepage requirements for high-standard cut-off walls. The research findings provide efficient and reliable chemical improvement solutions and a theoretical basis for anti-seepage engineering in foundation pits under complex coastal geological conditions.

## Introduction

Excavation engineering is crucial for ensuring the structural safety of buildings, and with the development of society, the scale of excavation engineering is also increasing [1,2]. In China’s coastal areas, the soil has a high water content and high compressibility [3,4], and the soil is mostly silt or fine sand, which has strong permeability. In addition, the groundwater level in the coastal areas is high, and under high groundwater level conditions, it is easy to cause fine sand to undergo piping and flow soil and other permeation failure phenomena [5,6], which can easily induce engineering accidents [7,8]. Improving the anti-seepage performance of the soil in the coastal areas is of great significance for ensuring the construction of excavation engineering.

Chemical soil modifiers have shown significant potential in regulating the mechanical properties of soil, with mechanisms that include cementation reactions, pore filling, particle aggregation, and hydrophobic modification [9]. Traditional inorganic modifiers (such as lime and cement) generate cementitious products (such as C-S-H gel and ettringite) through hydration reactions, which can effectively increase soil strength and optimize pore structure [10]. Chen [11] et al. found that increasing the lime content can increase the triaxial strength of red clay by 28.2%, and revealed the synergistic improvement mechanism of lime cementation and pore reconstruction in soil through SEM and NMR techniques. Xiao [12] investigated the modification effect of glass fiber-lime composite on red clay, and found that glass fiber forms a fibrous network structure inside the soil, enhancing the bonding at the soil-reinforcement interface and thereby strengthening the overall soil strength. Vinod [13] et al. revealed that calcium lignosulfonate modifiers can flocculate soil particles, thereby increasing the bonding between particles. The cement-peat ash composite system achieves pore blockage through the accumulation of cementitious products, significantly increasing the unconfined compressive strength under the optimal ratio [14].

In terms of anti-seepage performance improvement, superhydrophobic materials (such as organosilanes) can reduce the permeability coefficient of loess by 90% by changing the soil surface wettability, with mechanisms involving both increased contact angle and dual optimization of pore structure [15]. In addition, the introduction of polymers (such as PAM) can effectively improve the plastic flow of coarse-grained soil and extend the anti-seepage stability period, achieving a permeability coefficient of less than 10−5 m/s over the long term through particle aggregation and foam stability regulation [16].

Ding [17] carried out anti-seepage performance improvement on the waterproof curtain of silt strata, which can reduce the permeability coefficient to the order of 1 × 10−6 cm/s. However, their research did not consider the dissolution effect of cementitious phases in coastal fine sand soil caused by high-concentration Cl- erosion, leading to doubts about the durability of the improved system in seawater environments. Yang et al. [18] improved the balance shield mud in the water-rich gravel strata, but their conclusion that a certain permeability stability can be guaranteed when the hydraulic gradient is less than 19 still needs to be verified for its applicability to fine sand soil. In addition, the unique particle size distribution of fine sand soil (particles with a size of 0.125–0.5 mm account for more than 50%) results in significantly higher pore connectivity than that of clay [19], making it difficult for conventional soil modifiers, which lack fine particles, to form a continuous cementation network. Although the microbially induced calcium carbonate precipitation (MICP) technology can increase the soil cohesion by 40% [20], it relies on the migration mechanism of microorganisms, which is prone to uneven bio-clogging in the highly permeable medium of fine sand soil.

However, traditional chemical stabilizers (such as cement, lime, etc.) may be accompanied by potential ecological risks during their production and use, including high energy consumption, carbon emissions, or alterations to soil physicochemical properties. Hence, exploring environmentally friendly and sustainable soil improvement solutions has become a significant research trend. In this context, a range of eco-friendly biopolymer stabilizers have garnered widespread attention due to their renewable, biodegradable, and low environmental impact characteristics. Irem [21] modified kaolin using xanthan gum, guar gum, and polyacrylamide. The study found that guar gum can form a thick fibrous gel network, enabling the stabilized soil to exhibit excellent durability and resistance to freeze-thaw cycles. Zhang [22] revealed the microscopic mechanism of xanthan gum through scanning electron microscopy (SEM) and particle image velocimetry (PIV) techniques, demonstrating its ability to bind with water and form hydrogels, effectively cementing and filling sand pores. This significantly reduces the permeability of silty soil, enhances erosion resistance [23], and improves the water retention capacity of loess [24]. These studies provide strong support for the application of biopolymers in soil stabilization within environmentally sensitive areas.

The above research findings indicate that chemical soil modifiers have a good effect on improving the mechanical properties of soil. However, most existing studies focus on cohesive soil or coarse-grained soil systems. Fine sand soil in coastal areas has the characteristics of high permeability and low cohesion, and there is currently insufficient research on the improvement mechanisms of fine sand soil, especially in terms of improving the anti-seepage performance of fine sand soil. Therefore, this paper uses a soil seepage test system designed independently, selects the original soil from the Badagang Shiplock excavation in Zhejiang Province for seepage tests, analyzes the seepage erosion laws of fine sand soil under the action of flowing water, and explores the improvement effects of different types of chemical soil modifiers, different mixing amounts, and different curing times on fine sand soil.

## Soil seepage test

### Test system

**Fig 1:**
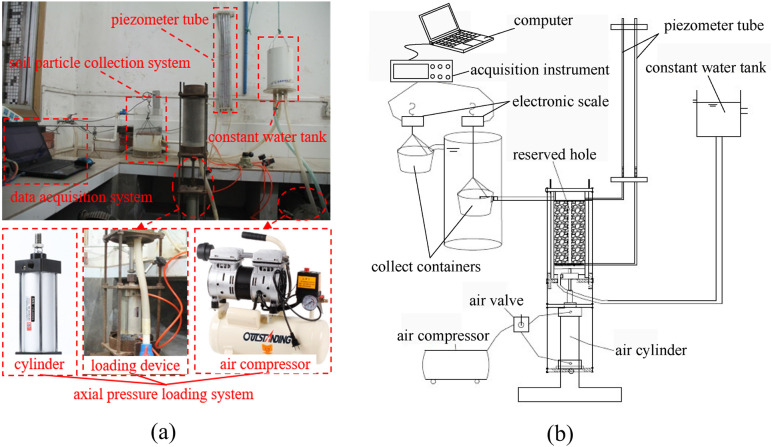
Schematic diagram of the test system. (a) Physical picture. (b) Schematic diagram.

The test employs a self-designed soil seepage test system [25], which mainly consists of a soil seepage cylinder, an axial pressure loading system, a soil particle collection device, and a constant water level simulation system, as shown in Fig 1. During the test, a stable seepage field in the test pipeline is first maintained by the constant water level tank, while the axial loading system applies a stable axial pressure to the sample. The seepage liquid, after permeating through the sample, passes through the downstream filtration system and enters the soil particle collection device equipped with a weighing sensor. After the seepage velocity reaches dynamic equilibrium, the water head difference between the upstream and downstream piezometers is measured using a high-precision differential pressure sensor. To accurately determine the termination condition of soil particle loss, a high-sensitivity mass sensor is integrated into the collection device to build a real-time monitoring system. When the monitoring data stabilizes (with a fluctuation amplitude of ≤0.1%), the termination of soil particle loss from the sample is determined.

During the test, the upstream water head was controlled by a constant water level device. All seepage tests were conducted in a constant-temperature laboratory environment, with the room temperature maintained at 20 ± 1°C to eliminate potential effects of temperature variations on water viscosity and the seepage process. The electronic balance in the soil particle collection device was connected to a computer, and the data acquisition frequency was set at 2 Hz, ensuring the continuity and high precision of the erosion mass data.

The mass inside the collection bucket is measured by the sensor in the soil particle collection device, and the weight of the soil particles in the collection bucket can be calculated using the following formula:


$$m_{s}=\frac{\gamma_{s}}{\gamma_{s}-\gamma_{w}}m_{b}$$ (1)

where *m*_*s*_ is the dry weight of the cumulative eroded soil mass (i.e., the actual mass of soil particles in a dry state), kg; *m*_*b*_ is the buoyant weight of the cumulative eroded soil mass (i.e., the apparent mass of soil particles measured by the sensor in water), kg; *γ*_*s*_ is the unit weight of soil particles, kN/m³; *γ*_*w*_ is the unit weight of water, kN/m³.

During the test, the water flow discharges through the upper outlet into the water measurement bucket. The seepage flow rate *Q* can be calculated based on the time and the change in the mass of water in the bucket. Combined with Darcy’s law, the corresponding soil permeability coefficient *k* can be determined.

In the test, the formula for calculating the seepage flow is as follows:


$$Q=\frac{\Delta G_{w}}{\Delta tg\rho_{w}}$$ (2)

where *Q* is the seepage flow rate, m³/s; *ΔG*_*w*_ is the weight of water discharged during time interval *Δ*t**, N; *ρ*_*w*_ is the density of water, kg/m³.

According to Darcy’s law, the seepage flow rate *Q* is proportional to the upstream-downstream head difference (*Δ*h**) and the cross-sectional area *A* perpendicular to the flow direction, and inversely proportional to the seepage path length *L*. Its expression is:


Q=k·i·A (3)

where *Q* is the seepage flow rate per unit time, m³/s; *k* is the soil permeability coefficient, cm/s; *i* is the hydraulic gradient, defined as the head loss per unit seepage path length, where **I* *= Δ*h*/*L*; *A* is the cross-sectional area of the specimen, m².

Substituting *i* = Δ*h*/*L* into Darcy’s law (3), the formula for calculating the permeability coefficient *k* is derived as follows:


$$k=\frac{QL}{A\cdot \Delta h}$$ (4)

where *k* is the soil permeability coefficient, cm/s; *Q* is the seepage flow rate per unit time, m³/s; *L* is the length of the specimen, cm; *A* is the cross-sectional area of the specimen, m²; Δ*h* is the upstream-downstream head difference, m.

### Preparation of test soil samples

**Fig 2:**
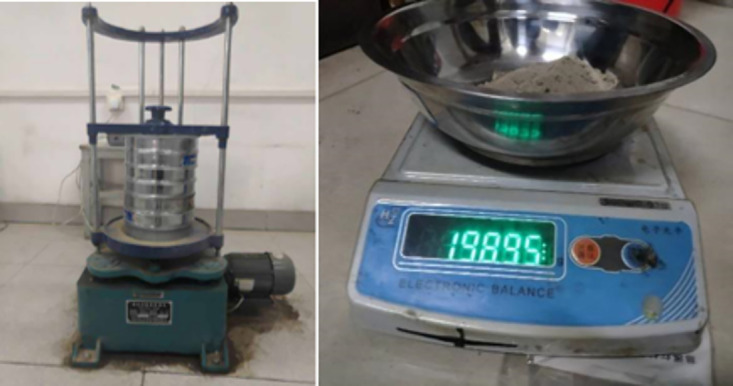
Sieve analysis test process.

**Fig 3:**
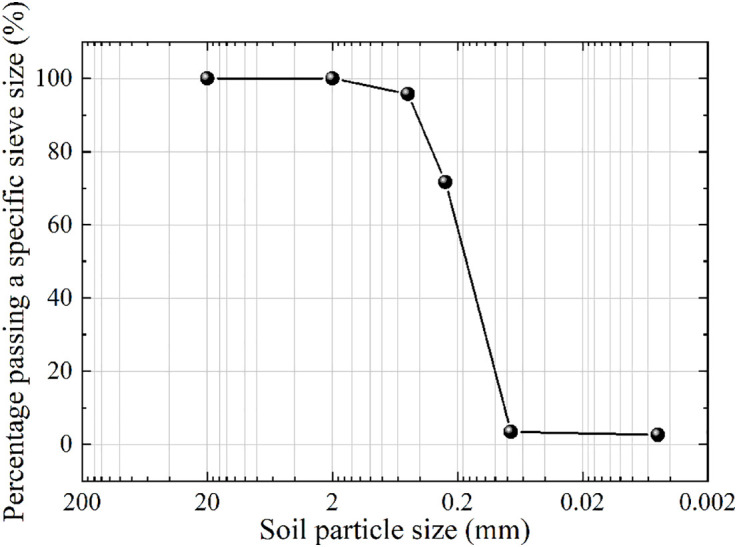
Particle size distribution curve of tested soil.

**Fig 4:**
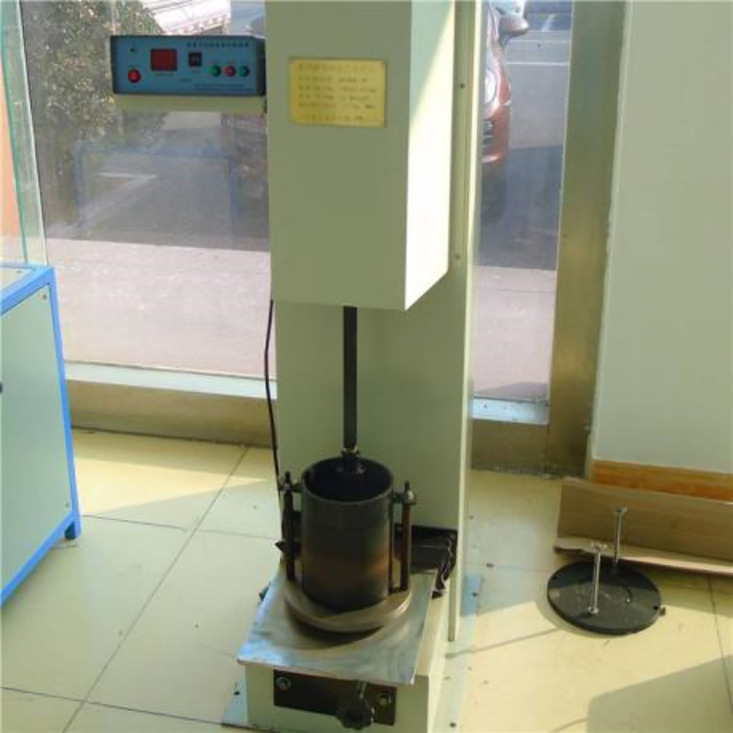
Compaction test process.

**Fig 5:**
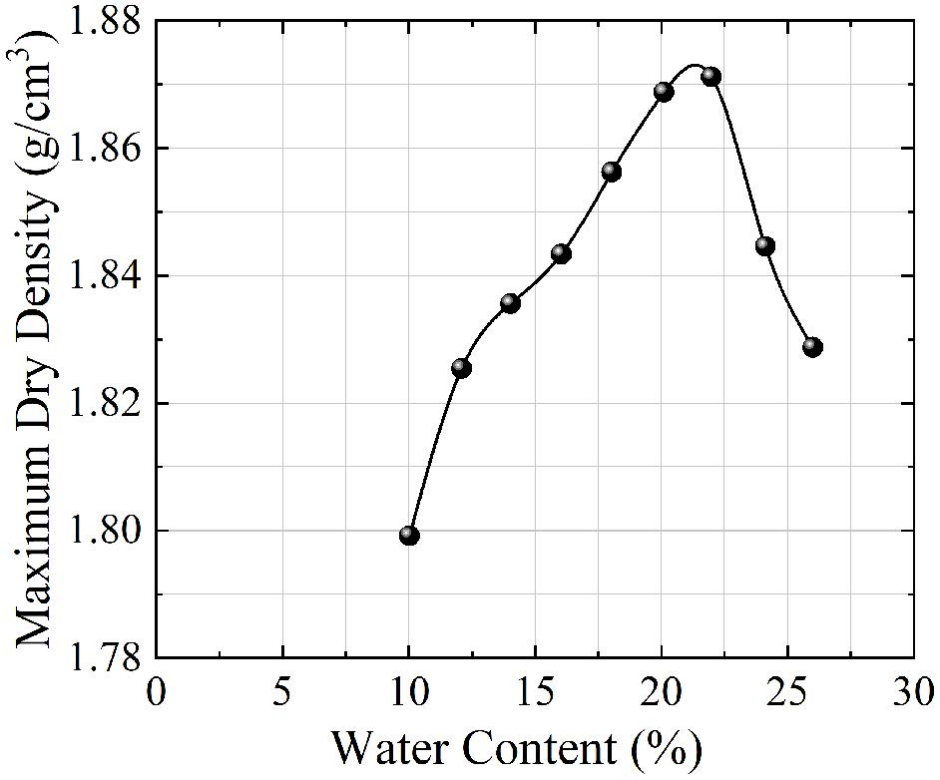
Relationship between maximum dry density and moisture content of test soil.

The soil material used in this test is the original soil from the Badagang Shiplock excavation in Zhejiang Province. After placing the original soil sample in a constant-temperature drying oven and drying it to a constant weight, the standard sieving method was used to remove coarse particles with a size greater than 50 mm (Fig 2), retaining the fine-grained soil components that are representative of the engineering context. The particle size distribution curve of the test soil is shown in Fig 3. The original data can be found in S1 Fig. Based on the particle size distribution characteristics of the on-site fine sand soil, the samples were reconstructed according to the mass percentage to ensure that the grading curve maintains geometric similarity with the naturally deposited soil body. The standard three-layer compaction method (Standard for Geotechnical Testing Methods [26]) was used for layered compaction (Fig 4) to eliminate structural differences in the soil body. Representative soil samples were taken from the central area of the compacted sample to measure its initial moisture content, as shown in Fig 5 (The original data can be found in S2 Table in S1 File). The maximum dry density of the test soil was measured to be 1.88 g/cm³, with an optimal moisture content of 22.6%.

### Test Scheme

In this test, the soil samples were chemically improved using a mixture of sodium silicate and aluminum dihydrogen phosphate solution, calcium lignosulfonate, and sodium polyacrylate reagents, and seepage tests were conducted on the improved samples. Based on the Hangzhou Babao Ship Lock foundation pit project, the vertical pressure on the soil under self-weight stress and additional loads generally ranges from 50 to 120 kPa. An intermediate value of 80 kPa was selected to simulate the stress state under real working conditions. Additionally, to ensure that significant seepage failure could be observed within a reasonable timeframe while avoiding excessively rapid failure due to an overly high gradient that would hinder the evaluation of the stabilizers’ effectiveness, a hydraulic gradient of 2.34 was adopted for this study.

Preliminary experiments indicated that the improvement effects of the selected chemical stabilizers tended to stabilize after 6 hours of curing. Furthermore, increasing the dosage beyond 6% resulted in only marginal performance improvements while significantly raising costs. Therefore, a curing time range of 0–6 hours and a dosage range of 0%–6% were chosen to investigate the permeability patterns of the soil under different stabilizer types, dosages, and curing durations. The specific test conditions are summarized in Table 1.

**Table 1:** Test conditions of chemical modification reagents.

Test Reagents	Test Number	Incorporation Content (%)	Curing Time (h)
Sodium Silicate and Aluminum Dihydrogen Phosphate Mixture Reagent	SL-C-1	0	0
	SL-C-2	2	0
	SL-C-3	4	0
	SL-C-4	6	0
	SL-T-1	4	0
	SL-T-2	4	2
	SL-T-3	4	4
	SL-T-4	4	6
Calcium Lignosulfonate Reagent	MG-C-1	0	0
	MG-C-2	2	0
	MG-C-3	4	0
	MG-C-4	6	0
	MG-T-1	4	0
	MG-T-2	4	2
	MG-T-3	4	4
	MG-T-4	4	6
Sodium Polyacrylate Reagent	JN-C-1	0	0
	JN-C-2	2	0
	JN-C-3	4	0
	JN-C-4	6	0
	JN-T-1	4	0
	JN-T-2	4	2
	JN-T-3	4	4
	JN-T-4	4	6

## Analysis of test results

### Impermeability test of soil treated with sodium silicate reagent

#### Different mixing ratios.

Comparison tests were carried out according to the mixing ratios set in Table 1. It was found that the control group sample SL-C-1, which did not add any modifier, experienced the collapse of the soil structure in the bucket after 13 minutes of seepage erosion. In contrast, the three groups of samples with modifiers only had a small amount of soil particle loss and the soil structure remained intact under the same conditions.

**Fig 6:**
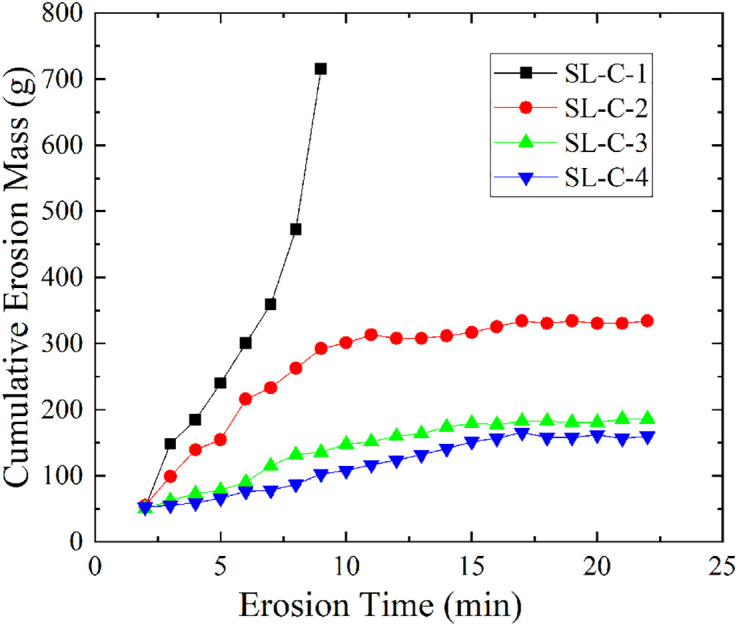
Variation curve of cumulative erosion mass versus time for SL-C group.

Fig 6 shows the relationship between the cumulative erosion mass and erosion time of samples SL-C-1 to SL-C-4 (S3 Table in S1 File provides the original data of Fig 6). Sample SL-C-1, under the action of seepage, rapidly lost the integrity of its soil structure, with its cumulative erosion mass increasing exponentially over time, indicating a continuously accelerating erosion rate. In contrast, samples SL-C-2 to SL-C-4, due to the addition of modifiers, exhibited a progressive trend in cumulative erosion mass that first increased and then gradually stabilized over time, with the stabilization time extending as the mixing ratio increased, at 10.8 min, 13.6 min, and 17.1 min, respectively. This indicates that the sodium silicate reagent delayed the erosion process of the soil. In terms of the final cumulative erosion mass, sample SL-C-1 was 742 g, while samples SL-C-2 to SL-C-4 were 306.43 g, 153.88g, and125.69g, respectively, with the cumulative erosion mass decreasing progressively. Additionally, the erosion rates of samples SL-C-1 to SL-C-4 were 89.39 g/min, 28.69 g/min, 11.86 g/min, and 5.93 g/min, respectively, further demonstrating that the higher the mixing ratio of sodium silicate, the better the protective effect on the soil structure.

The observed experimental phenomena can be explained by the chemical stabilization mechanism of sodium silicate. Through hydrolysis and polymerization reactions (see Reaction Equations I ~ IV), sodium silicate generates active silicic acid gel, which effectively cements soil particles and forms a dense protective layer both within the soil matrix and on its surface. This process not only enhances interparticle cohesion but, more importantly, significantly improves the soil’s impermeability by clogging pore channels [27]. Thus, the gel structure formed by sodium silicate effectively binds soil particles and fills pores [28], consistent with the experimental results showing significantly reduced erosion rates and maintained structural integrity of the stabilized soil.

Ⅰ. Hydrolysis Reaction:


$$Na_{\text{2}}O\cdot nSiO_{\text{2}}+mH_{\text{2}}O\to 2NaOH+nSiO_{\text{2}}\cdot (m-1)H_{\text{2}}O$$

Ⅱ. Polymerization Reaction:


$$Na_{\text{2}}O\cdot nSiO_{\text{2}}+CO_{\text{2}}+xH_{\text{2}}O\to Na_{\text{2}}CO_{\text{3}}+nSiO_{\text{2}}\cdot xH_{\text{2}}O$$

Ⅲ. Adding Aluminum Dihydrogen Phosphate to Enhance Bonding Strength:


$$Na_{\text{2}}O\cdot nSiO_{\text{2}}+\text{2}H^{+}+xH_{\text{2}}O\to 2Na^{+}+nSiO_{\text{2}}\cdot (x+1)H_{\text{2}}O$$

Ⅳ. Reaction of sodium silicate with Aluminum Ions:


$$3Na_{\text{2}}O\cdot nSiO_{\text{2}}\cdot H_{\text{2}}O+2Al^{3+}\to 6Na^{+}+2Al(OH{)}_{\text{3}}+nSiO_{\text{2}}\cdot (m-3)H_{\text{2}}O$$

#### Different curing times.

**Fig 7:**
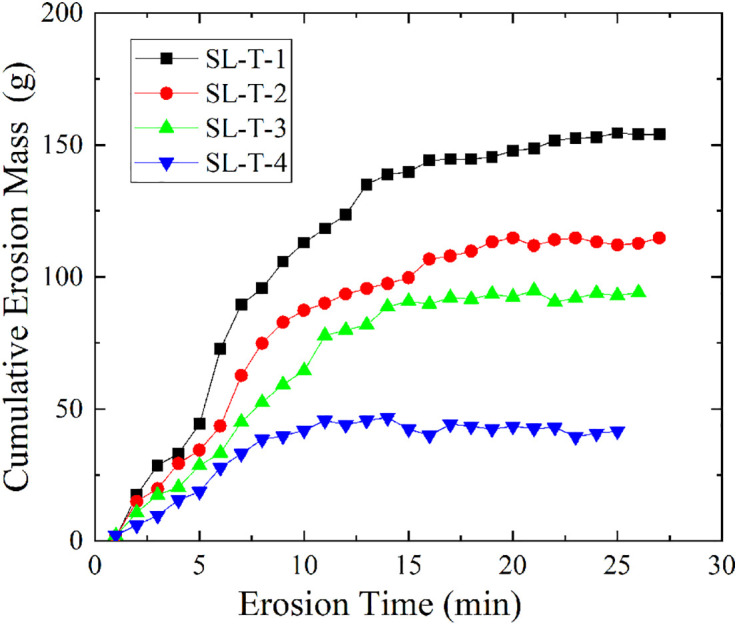
Variation curves of SL-T group with different curing times.

Fig 7 shows the changes in cumulative erosion mass of soil samples treated with sodium silicate reagent under different curing times (S4 Table in S1 File provides the original data of Fig 7). The cumulative erosion mass of the four groups of samples SL-T-1 to SL-T-4 all showed a trend of first increasing and then gradually stabilizing as the curing time increased. Specifically, the erosion mass of sample SL-T-1 increased rapidly in the early stage, but its growth rate gradually slowed down as the curing time extended, and it reached a stable state at 20.5 min, with a final cumulative erosion mass of 153.98 g. Samples SL-T-2, SL-T-3, and SL-T-4 reached stable states at 19.6 min, 18.2 min, and 12.1 min, respectively, with cumulative erosion masses of 114.86 g, 94.35 g, and 42.21 g, respectively. This indicates that as the curing time increases, the soil samples can reach a stable state more quickly, and the cumulative erosion mass gradually decreases, demonstrating that the anti-seepage improvement effect of the soil significantly increases with the extended curing time. Further analysis revealed that the erosion rates of samples SL-T-1 to SL-T-4 were 7.51 g/min, 5.86 g/min, 5.18 g/min, and 3.49 g/min, respectively, with the erosion rate gradually decreasing as the curing time increased. This further confirms that a longer curing time helps enhance the soil’s anti-erosion properties. The gelation reaction of sodium silicate in the soil requires a certain amount of time to proceed fully. As the curing time increases, the reaction between sodium silicate and soil particles becomes more complete, forming a denser cementation network, thereby significantly improving the soil’s structural stability and scour resistance, and effectively reducing soil particle loss.

### Impermeability test of soil treated with calcium lignosulfonate reagent

#### Different mixing ratios.

**Fig 8:**
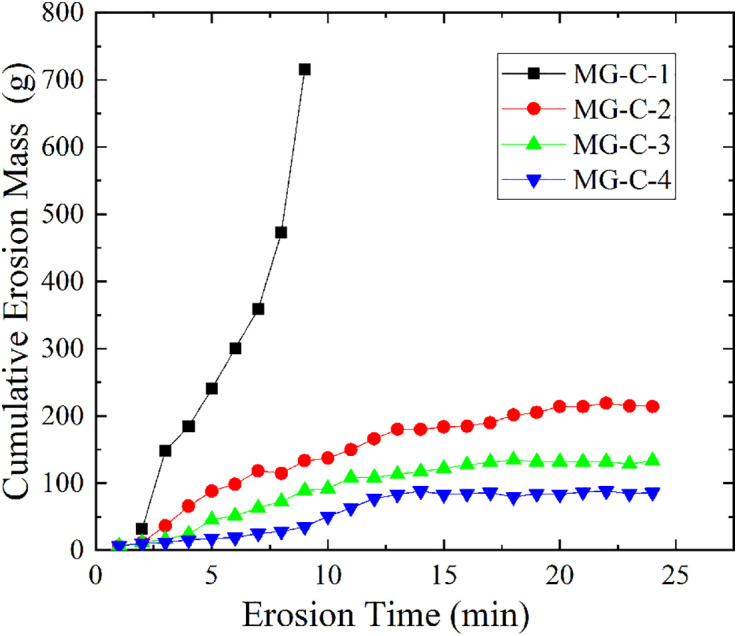
Variation curve of cumulative erosion mass versus time for MG-C group.

Fig 8 shows the relationship between different mixing ratios of calcium lignosulfonate and the cumulative erosion mass of soil samples (S5 Table in S1 File provides the original data of Fig 8). Sample MG-C-1, which did not have any modifier added, experienced rapid soil structure destruction under seepage action, with its cumulative erosion mass increasing exponentially over time, eventually reaching 742 g. This indicates a continuously accelerating erosion rate and extremely poor soil erosion resistance. In contrast, samples MG-C-2, MG-C-3, and MG-C-4, with modifiers added, all showed a trend of first increasing and then gradually stabilizing in cumulative erosion mass over time, with final cumulative erosion masses of 215.12 g, 133.5 g, and 86.30 g, respectively. This indicates that the higher the mixing ratio, the stronger the soil’s erosion resistance. Further analysis revealed that the erosion times for samples MG-C-2 to MG-C-4 to reach a stable state were 19.6 min, 17.4 min, and 13.3 min, respectively. That is, as the mixing ratio increased, the time for the samples to reach a stable state gradually shortened, and the corresponding erosion rates also decreased progressively, at 13.24 g/min, 9.559 g/min, and 5.31 g/min, respectively. This shows that increasing the mixing ratio of calcium lignosulfonate can accelerate the soil sample’s reaching of a stable erosion state and effectively suppress soil erosion development.

The observed improvement can be attributed to the stabilization mechanism of calcium lignosulfonate. Its molecules are rich in hydroxyl and sulfonic acid groups, which enable chemical adsorption onto soil particles and form a network-like cementation structure on their surfaces, thereby reconstructing the soil skeleton. On one hand, this network enhances mechanical interlocking between particles through bridging effects; on the other hand, it optimizes pore structure via steric hindrance effects, ultimately significantly improving the soil’s impermeability [29]. Consequently, the stable network structure formed by calcium lignosulfonate effectively enhances the connectivity between soil particles and the compactness of the system, which aligns well with the experimental observations of reduced erosion mass, shortened stabilization time, and improved erosion resistance with increasing dosage.

#### Different curing times.

**Fig 9:**
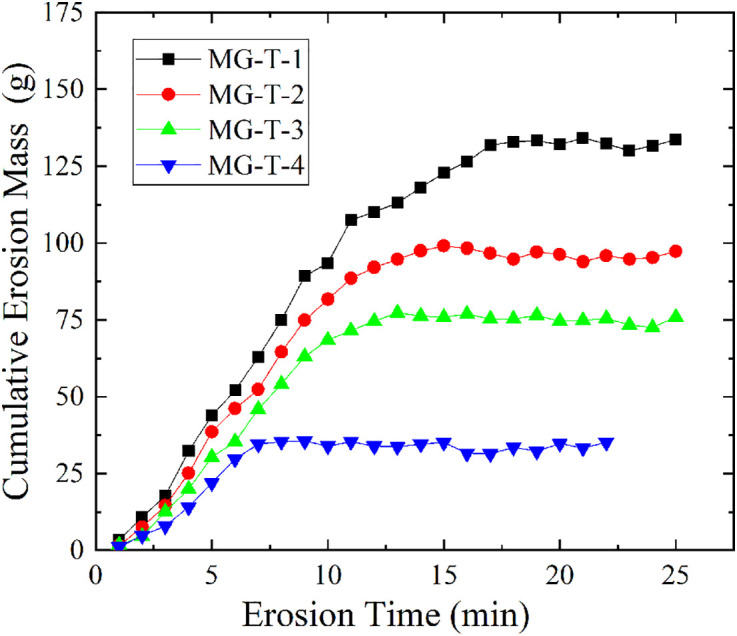
Variation curves of MG-T group with different curing times.

Fig 9 reveals the relationship between the cumulative erosion mass and erosion time of soil samples under different curing times (S6 Table in S1 File provides the original data of Fig 9). As the curing time increases, the final cumulative erosion mass of the samples gradually decreases, being 133.50 g, 97.68 g, 76.12 g, and 35.40 g, respectively. This indicates that a longer curing time helps enhance the soil’s erosion resistance and reduce soil particle loss. Further analysis shows that the erosion time for the four groups of samples to reach seepage stability decreases as the curing time increases, at 15.8 min, 14.4 min, 12.1 min, and 5.6 min, respectively. This suggests that the longer the curing time, the faster the soil samples can reach seepage stability. This is because the chemical reaction of calcium lignosulfonate in the soil requires a certain amount of time to proceed fully. As the curing time extends, its combination with soil particles becomes more secure, and the formed network structure becomes denser, thereby significantly improving the soil’s structural stability and scour resistance. Although the erosion rates of the four groups of samples are 8.45 g/min, 6.78 g/min, 6.29 g/min, and 6.32 g/min, with some fluctuations, the overall trend still shows that a longer curing time helps reduce the erosion rate, further verifying the positive impact of curing time on the anti-seepage performance of soil improved by calcium lignosulfonate.

### Impermeability test of soil treated with sodium polyacrylate reagent

#### Different mixing ratios.

**Fig 10:**
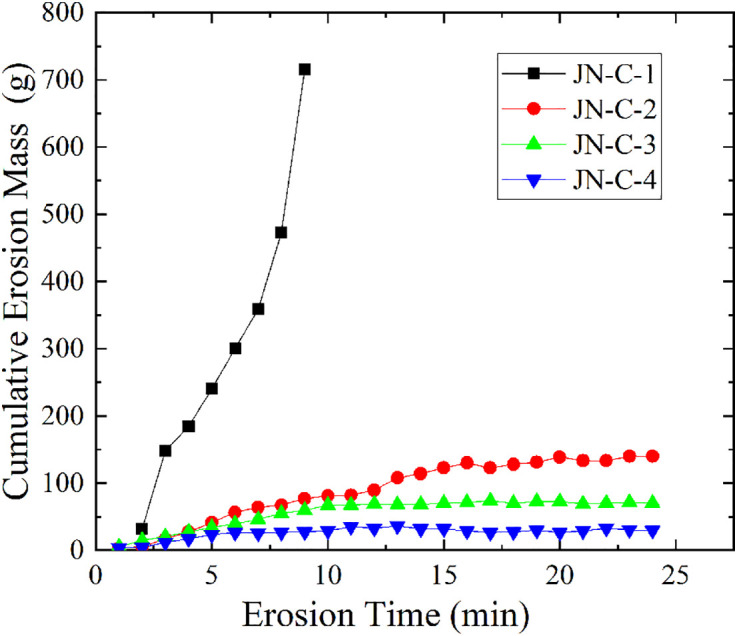
Variation curve of cumulative erosion mass versus time for JN-C group.

Fig 10 shows the effect of different mixing ratios of sodium polyacrylate on the cumulative erosion mass of soil (S7 Table in S1 File provides the original data of Fig 10). Sample JN-C-1, which did not have any modifier added, experienced rapid soil structure destruction under seepage action, with its cumulative erosion mass increasing exponentially over time. In contrast, samples JN-C-2, JN-C-3, and JN-C-4, with sodium polyacrylate added, all showed a trend of first increasing and then gradually stabilizing in cumulative erosion mass over time, with final cumulative erosion masses of 143.2 g, 72.9 g, and 35.0 g, respectively. This indicates that the higher the mixing ratio, the stronger the soil’s erosion resistance. Further analysis revealed that the erosion times for samples JN-C-2 to JN-C-4 to reach a stable state were 12.1 min and 6.3 min, respectively. That is, as the mixing ratio increased, the time for the samples to reach a stable state gradually shortened, and the corresponding erosion rates also decreased progressively, at 7.93 g/min, 4.84 g/min, and 3.98 g/min, respectively. This shows that increasing the mixing ratio of sodium polyacrylate can accelerate the soil sample’s reaching of a stable erosion state and effectively suppress soil erosion development.

Sodium polyacrylate can form hydrogen bonds [30] between the carboxyl groups on its molecular chains and the hydroxyl groups on the surface of soil particles, promoting the agglomeration of clay minerals into stable aggregate structures, thereby enhancing the overall integrity and stability of the soil structure [31]. Consequently, the aggregation structure facilitated by hydrogen bonding effectively strengthens the inter-particle connectivity and compactness, leading to a significant reduction in erosion mass, shortened stabilization time, and improved impermeability of the treated soil in the experiments.

#### Different curing times.

**Fig 11:**
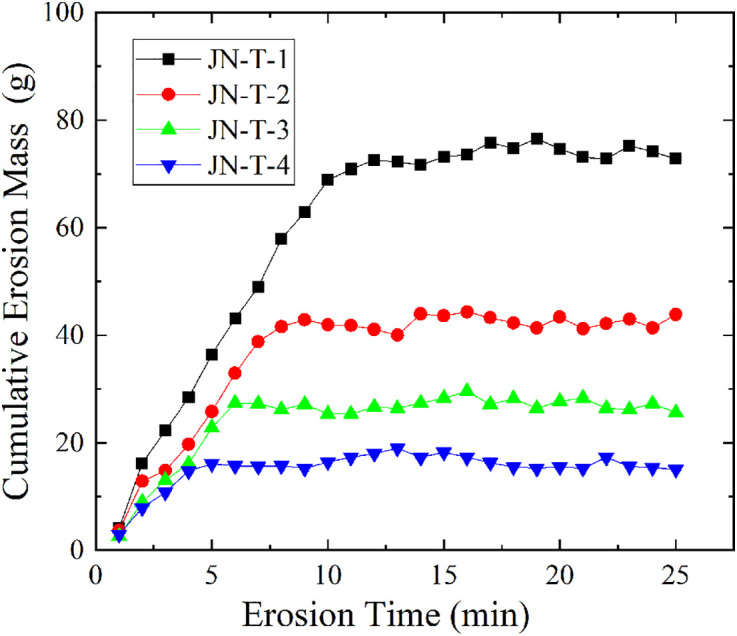
Variation curve of cumulative erosion mass versus time for JN-T group.

Figure 11 shows the changes in cumulative erosion mass of soil samples under different curing times (S8 Table in S1 File provides the original data of Fig 11). As can be seen from the figure, the final cumulative erosion masses of samples JN-T-1 to JN-T-4 are 72.90g, 44.00g, 25.69g, and 15.21g, respectively. Sample JN-T-1 experienced rapid initial erosion mass growth, but its growth rate gradually slowed down as the curing time extended, reaching a stable state at 12.3 min. Samples JN-T-2, JN-T-3, and JN-T-4 reached stable states at 8.2 min, 5.7 min, and 4.1 min, respectively, with decreasing cumulative erosion mass. This indicates that as the curing time increases, the erosion resistance of the soil samples gradually enhances, allowing them to more effectively withstand the scouring action of seepage. With the extended curing time, the reaction between sodium polyacrylate and soil particles becomes more complete, forming tighter aggregate structures and thus stronger erosion resistance. Therefore, prolonging the curing time helps further improve the anti-seepage performance of soil modified by sodium polyacrylate.

### Comparative analysis of amelioration effects by different stabilizer types

**Fig 12:**
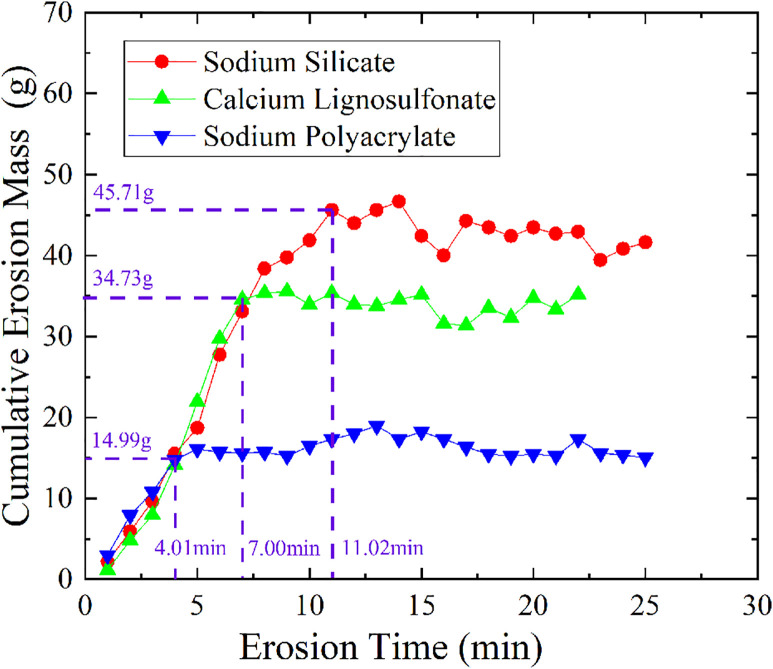
Variation curves of cumulative erosion mass with time for the three stabilizers.

The above test results show that the three chemical soil modifiers all have a good effect on improving the anti-seepage performance of soil. To compare and analyze the differences in the improvement effects of the three chemical stabilizers on soil impermeability, the relationship between cumulative erosion mass and time for soil samples treated with each stabilizer under the same dosage (4%) and curing time (6 h) is plotted in Fig 12 (S9 Table in S1 File provides the original data of Fig 12). As shown in the figure, the cumulative erosion mass of soil treated with sodium polyacrylate is significantly lower than that of soil treated with sodium silicate and calcium lignosulfonate. Specifically, the erosion mass of sodium polyacrylate–treated soil stabilizes at 4.01 min, with a cumulative erosion mass of 14.99 g. For calcium lignosulfonate–treated soil, stabilization occurs at 7.00 min, with a cumulative erosion mass of 34.73 g. In contrast, sodium silicate–treated soil requires 11.02 min to stabilize, and its cumulative erosion mass is as high as 45.71 g. These results indicate that sodium polyacrylate–treated soil exhibits superior impermeability.

In terms of the mechanism, sodium polyacrylate can strongly bind to soil particles through chelation, effectively immobilizing free metal ions in the soil and reducing their mobility, while simultaneously forming a cross-linked network structure that enhances soil density [32]. In comparison, sodium silicate primarily relies on silicic acid gel to fill pores, and although calcium lignosulfonate has some complexation ability, its chelation strength and stability are far inferior to those of sodium polyacrylate, making it unable to form an equally dense network. Additionally, sodium polyacrylate can bind to hydroxyl groups on the surface of soil particles via hydrogen bonds, promoting the formation of aggregate structures [33]. This significantly alters the particle size distribution of the soil, reduces the proportion of fine pores, and thereby decreases the connectivity of seepage channels [34]. In contrast, the cementitious structure formed by sodium silicate is prone to shrinkage and microcracking, while the cementation effect of calcium lignosulfonate is limited by the completeness of the reaction, resulting in poor uniformity and continuity [35,36]. Therefore, through a multi-scale synergistic improvement mechanism, sodium polyacrylate achieves efficient pore sealing and structural stability in the soil, and its impermeability improvement effect is significantly superior to that of sodium silicate and calcium lignosulfonate.

**Fig 13:**
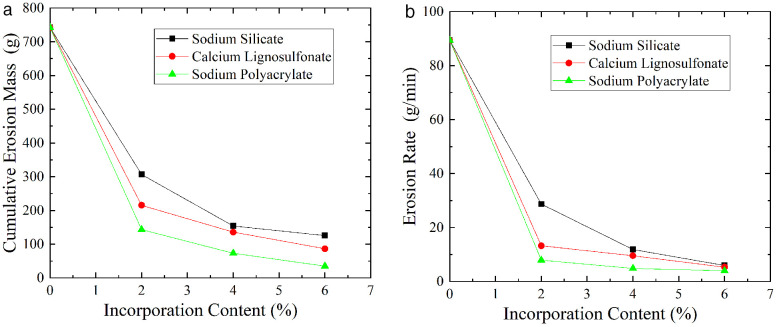
Variation relationship of soil cumulative erosion mass and erosion rate with incorporation content. (a) Cumulative erosion mass-incorporation content. (b) Erosion rate-incorporation content.

Figure 13 shows the relationship between the cumulative erosion mass and erosion rate of soil with three types of chemical modifiers as the mixing ratio changes (S10 Table in S1 File provides the original data of Fig 13). It can be seen from Figure 13 that both the cumulative erosion mass and erosion rate of soil decrease with the increase of the mixing ratio of modifiers. Compared with the soil without modifiers, the degree of improvement is the greatest when the mixing ratio is 2%, and the degree of improvement gradually decreases with the increase of the mixing ratio. Among the three modifiers, under the same mixing ratio, the cumulative erosion mass and erosion rate of soil modified by sodium polyacrylate are significantly lower than those of the other two modifiers.

**Fig 14:**
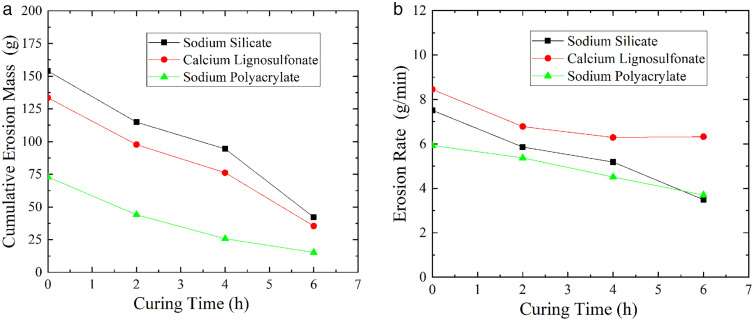
Variation relationship of soil cumulative erosion mass and erosion rate with curing time. (a) Cumulative erosion mass-curing time. (b) Erosion rate-curing time.

Figure 14 shows the relationship between the cumulative erosion mass and erosion rate of soil modified by the three chemical modifiers as the curing time changes (S11 Table in S1 File provides the original data of Fig 14). It was found that both the cumulative erosion mass and erosion rate gradually decreased with the increase of curing time. When the curing time was 2 hours, the cumulative erosion masses of the silty sand treated with sodium silicate, calcium lignosulfonate, and sodium polyacrylate were 114.86 g, 97.68 g, and 44 g, respectively. Compared to the untreated soil, the erosion masses were reduced by 627.14 g, 644.32 g, and 698 g, respectively. When the curing time was extended to 6 hours, the cumulative erosion masses of the soils treated with sodium silicate, calcium lignosulfonate, and sodium polyacrylate were 42.21 g, 35.4 g, and 15.21 g. Compared to the untreated soil, the erosion masses were reduced by 699.79 g, 706.6 g, and 726.79 g.

Furthermore, comparing the improvement effects of the three chemical stabilizers at different curing times revealed that the effectiveness of sodium silicate at 2 hours of curing was approximately 89.62% of that achieved at 6 hours. For calcium lignosulfonate, the effectiveness at 2 hours of curing was about 91.18% of that at 6 hours. In contrast, sodium polyacrylate reached approximately 96.10% of its improvement effectiveness at 6 hours after only 2 hours of curing. These results indicate that sodium silicate and calcium lignosulfonate require longer curing times to fully exert their improvement effects, whereas sodium polyacrylate achieves most of its impermeability improvement within a shorter curing period.

In addition, when the mixing ratio of chemical modifiers and the curing time of samples are the same, the improvement effects of sodium silicate mixture, calcium phosphate reagent, and sodium polyacrylate reagent on soil are compared and analyzed. The permeability coefficient of the soil samples is calculated according to Equation (4), and the comparison results are shown in Table 2. Among the three types of modifiers, the soil samples modified by sodium polyacrylate reagent have the smallest permeability coefficient, erosion rate, and cumulative erosion mass.

**Table 2:** Comparative analysis of test results.

Test Conditions		Cumulative Erosion Mass (g)	Stabilization Time (min)	Average Erosion Rate (g/min)	Permeability Coefficient (cm/s)
Sodium Silicate Mixture	SL-C-1	742.00	8.3	89.39	4.32 × 10^−4^
	SL-C-2	306.43	10.8	28.69	6.85 × 10^−5^
	SL-C-3	153.88	13.6	11.86	4.31 × 10^−5^
	SL-C-4	125.69	17.1	5.93	3.22 × 10^−5^
Calcium Lignosulfonate	MG-C-1	742.00	8.3	89.39	4.32 × 10^−4^
	MG-C-2	215.12	19.6	13.24	5.33 × 10^−5^
	MG-C-3	133.50	17.4	9.55	3.66 × 10^−6^
	MG-C-4	86.30	13.3	5.31	2.18 × 10^−6^
Sodium Polyacrylate	JN-C-1	742.00	8.3	89.39	4.32 × 10^−4^
	JN-C-2	143.56	15.4	7.93	5.41 × 10^−6^
	JN-C-3	72.90	12.1	4.84	4.36 × 10^−7^
	JN-C-4	35.00	6.3	3.98	3.15 × 10^−7^

In accordance with the ‘Design Code for Levee Projects (GB 50286-2013)’ and relevant engineering practices, the permeability coefficient of soil materials used for anti-seepage purposes is generally required to be below 10 ⁻ ⁵ cm/s, while for high-standard foundation pit cut-off walls, the permeability coefficient must meet the requirement of 10 ⁻ ⁶ cm/s [37,38]. The test results of this study demonstrate that the permeability of silty sand treated with sodium polyacrylate satisfies these engineering anti-seepage requirements.

From an economic perspective, the unit costs of the stabilizers are as follows: sodium silicate at 10.38 USD/kg, calcium lignosulfonate at 12.62 USD/kg, and sodium polyacrylate at 3.50 USD/kg. Overall, sodium polyacrylate is the most cost-effective option for stabilizing silty sand.

Taking into account the improvement effect, curing time, and economy, sodium polyacrylate has a significant advantage in enhancing the soil’s anti-seepage performance, especially for scenarios that require rapid construction. The appropriate modifier, its mixing ratio, and curing time can be selected based on specific needs and conditions to achieve the best anti-seepage effect of the soil.

## Conclusions

This study investigated the effectiveness of three chemical stabilizers—sodium silicate, calcium lignosulfonate, and sodium polyacrylate—in improving the impermeability of coastal silty sand using a self-designed seepage testing system. The main conclusions are as follows:

1. The cumulative erosion mass of the untreated soil increased exponentially over time, with an erosion rate as high as 89.39 g/min. In contrast, the cumulative erosion mass of silty sand treated with any of the three chemical stabilizers exhibited a trend of initial increase followed by stabilization, and the erosion rates were all reduced to below 30 g/min.
2. The impermeability improvement effects of all three stabilizers increased with higher dosages. However, sodium polyacrylate achieved significant improvement even at low dosages (4%–6%). Curing time had a considerable impact on the effectiveness of sodium silicate and calcium lignosulfonate, whereas sodium polyacrylate reached 96.10% of its maximum impermeability improvement after just 2 hours of curing.
3. Under the conditions of 4% dosage and 6 hours of curing, sodium polyacrylate reduced the erosion rate to 3.98 g/min, limited the cumulative erosion mass to only 35 g, stabilized the permeability coefficient within the range of 10 ⁻ ⁶ to 10 ⁻ ⁷ cm/s, and shortened the permeability stabilization time to 6.3 minutes. Its overall improvement effect was superior to that of the other two stabilizers.

This study confirms the comprehensive advantages of sodium polyacrylate in enhancing the impermeability of silty sand. Its efficiency and stability make it suitable for anti-seepage engineering in coastal areas with high water levels, demonstrating significant practical application value. Future work may involve microstructural testing techniques to analyze the cementation products and pore structure evolution of stabilized silty sand, further elucidating the mechanisms of chemical stabilizers. Additionally, long-term field monitoring experiments could be conducted to evaluate the durability of these chemical stabilizers in real-service environments.

## Supporting information

S1 File **S1 Figure.** The original data in Fig 3. S2 Table. The original data in Fig 5. S3 Table. The original data in Fig 6.S4 Table. The original data in Fig 7. S5 Table. The original data in Fig 8. S6 Table. The original data in Fig 9. S7 Table. The original data in Fig 10. S8 Table. The original data in Fig 11. S9 Table. The original data in Fig 12. S10 Table. The original data in Fig 13. S11 Table. The original data in Fig 14.
(ZIP)
