# Correction: Experimental study on soil impermeability based on chemical improvement method

**DOI:** 10.1371/journal.pone.0339804

**Published:** 2025-12-31

**Authors:** Hai Shang, Bin Xiong, Chuan Jin, Deying Tang, Neng Xiong, Yifan Chen

The first author’s name is spelled incorrectly. The correct name is: Hai Shang. The correct citation is: Shang H, Xiong B, Jin C, Tang D, Xiong N, Chen Y (2025) Experimental study on soil impermeability based on chemical improvement method. PLoS One 20 (10): e0334100. https://doi.org/10.1371/journal.pone.0334100.

## References

[1] Sang H, Xiong B, Jin C, Tang D, Xiong N, Chen Y. Experimental study on soil impermeability based on chemical improvement method. PLoS One. 2025;20(10):e0334100. doi: 10.1371/journal.pone.0334100 41118373 PMC12539693

