# Supplementary material for: Experimental study on soil impermeability based on chemical improvement method
Source: PLoS One. 2025 Oct 21;20(10):e0334100. doi: 10.1371/journal.pone.0334100 (PMC12539693; doi:10.1371/journal.pone.0334100)
Supplement: S1 File — S1 Figure. The original data in Fig 3. S2 Table. The original data in Fig 5. S3 Table. The original data in Fig 6.S4 Table. The original data in Fig 7. S5 Table. The original data in Fig 8. S6 Table. The original data in Fig 9. S7 Table. The original data in Fig 10. S8 Table. The original data in Fig 11. S9 Table. The original data in Fig 12. S10 Table. The original data in Fig 13. S11 Table. The original data in Fig 14. (ZIP) [file pone.0334100.s001.zip › supporting information/S1 Figure. The original data in Fig 3 .pdf]

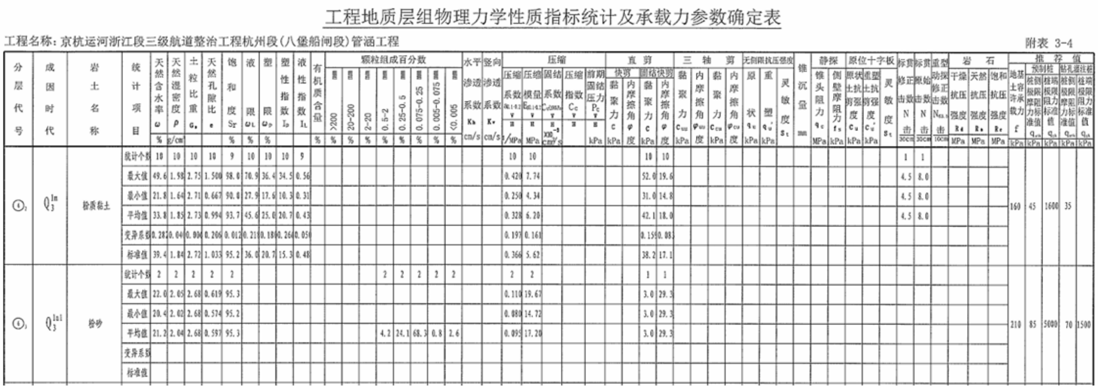

工程地质层组物理力学性质指标统计及承载力参数确定表

工程名称：京杭运河浙江段三级航道整治工程杭州段（八堡船闸段）管涵工程

附表 3-4

分层代号	成因时代	岩土名称	统计项目	天然含水率 ω %	天然湿密度 ρ g/cm³	土粒比重 G_s	天然孔隙比 e	饱和度 S_r %	液限 $ω_L$ %	塑限 $ω_P$ %	塑性指数 I_P	液性指数 I_L	有机质含量 %	颗粒组成百分数 >200 %	20-200 %	2-20 %	0.5-2 %	0.25-0.5 %	0.075-0.25 %	0.005-0.075 %	<0.005 %
④2	Q_3^{1m}	粉质黏土	统计个数	10	10	10	10	9	10	10	10	9									
			最大值	49.6	1.98	2.75	1.500	98.0	70.9	36.4	34.5	0.56									
			最小值	21.8	1.64	2.71	0.667	90.0	27.9	17.6	10.3	0.31									
			平均值	33.8	1.85	2.73	0.994	93.7	45.6	25.0	20.7	0.43									
			变异系数	0.282	0.040	0.004	0.206	0.012	0.219	0.180	0.264	0.050									
			标准值	39.4	1.84	2.72	1.033	95.2	36.0	20.7	15.3	0.48									
④3	Q_3^{1al}	粉砂	统计个数	2	2	2	2	2									2	2	2	2	2
			最大值	22.0	2.05	2.68	0.619	95.3													
			最小值	20.4	2.02	2.68	0.574	95.2													
			平均值	21.2	2.04	2.68	0.597	95.3									4.2	24.1	68.3	0.8	2.6
			变异系数																		
			标准值																		

分层代号	统计项目	水平渗透系数 K_h cm/s	竖向渗透系数 K_v cm/s	压缩 压缩系数 $a_{0.1-0.2}$ 1/MPa	压缩模量 $E_{s0.1-0.2}$ MPa	固结系数 C_v $×10^{-3}$ cm^2/s	压缩指数 C_c	前期固结压力 P_c kPa	直剪 快剪 黏聚力 c kPa	快剪 内摩擦角 φ 度	固结快剪 黏聚力 c kPa	固结快剪 内摩擦角 φ 度	三轴剪 黏聚力 c_{uu} kPa	内摩擦角 $φ_{uu}$ 度	黏聚力 c_{cu} kPa	内摩擦角 $φ_{cu}$ 度	无侧限抗压强度 原状 q_u kPa	重塑 q_u' kPa	灵敏度 S_t	锥沉量 mm
④2	统计个数			10	10						10	10								
	最大值			0.420	7.74						52.0	19.6								
	最小值			0.250	4.34						31.0	14.8								
	平均值			0.328	6.20						42.1	18.0								
	变异系数			0.197	0.161						0.159	0.083								
	标准值			0.366	5.62						38.2	17.1								
④3	统计个数			2	2						1	1								
	最大值			0.110	19.67						3.0	29.3								
	最小值			0.080	14.72						3.0	29.3								
	平均值			0.095	17.20						3.0	29.3								
	变异系数																			
	标准值																			

分层代号	统计项目	静探 锥头阻力 q_c MPa	侧壁摩阻力 f_s kPa	原位十字板 原状土抗剪强度 C_u kPa	重塑土抗剪强度 C_u' kPa	灵敏度 S_t	标贯修正击数 N 击/30cm	标贯原始击数 N 击/30cm	重型动探修正击数 $N_{63.5}$ 击/10cm	岩石 干燥抗压强度 R_d MPa	天然抗压强度 R_n MPa	饱和抗压强度 R_c MPa	推荐值 地基土容许承载力 f kPa	预制桩 桩侧极限摩阻力标准值 q_{sik} kPa	预制桩 桩端极限阻力标准值 q_{pk} kPa	钻孔灌注桩 桩侧极限摩阻力标准值 q_{sik} kPa	钻孔灌注桩 桩端极限阻力标准值 q_{pk} kPa
④2	统计个数						1	1					160	45	1600	35	
	最大值						4.5	8.0									
	最小值						4.5	8.0									
	平均值						4.5	8.0									
	变异系数																
	标准值																
④3	统计个数												210	85	5000	70	1500
	最大值																
	最小值																
	平均值																
	变异系数																
	标准值																

S1 Figure. The original data in Fig 3

S1 Table. The original data in Fig 3

Soil particle size (mm)	Percentage passing a specific sieve size (%)
0.005	2.6
0.075	3.4
0.25	71.7
0.5	95.8
2	100
20	100
