# Supplementary material for: Experimental study on soil impermeability based on chemical improvement method
Source: PLoS One. 2025 Oct 21;20(10):e0334100. doi: 10.1371/journal.pone.0334100 (PMC12539693; doi:10.1371/journal.pone.0334100)
Supplement: S1 File — S1 Figure. The original data in Fig 3. S2 Table. The original data in Fig 5. S3 Table. The original data in Fig 6.S4 Table. The original data in Fig 7. S5 Table. The original data in Fig 8. S6 Table. The original data in Fig 9. S7 Table. The original data in Fig 10. S8 Table. The original data in Fig 11. S9 Table. The original data in Fig 12. S10 Table. The original data in Fig 13. S11 Table. The original data in Fig 14. (ZIP) [file pone.0334100.s001.zip › supporting information/S11 Table. The original data in Fig 14.pdf]

S11 Table(a). The original data in Fig 14(a)

Curing Time (h)	Cumulative Erosion Mass (g)		
	Sodium Silicate	Calcium Lignosulfonate	Sodium Polyacrylate
0	153.98	133.5	72.9
2	114.86	97.68	44
4	94.35	76.12	25.69
6	42.21	35.4	15.21

S11 Table(b). The original data in Fig 14(b)

Curing Time (h)	Erosion Rate (g/min)		
	Sodium Silicate	Calcium Lignosulfonate	Sodium Polyacrylate
0	7.51	8.45	5.93
2	5.86	6.78	5.37
4	5.18	6.29	4.51
6	3.49	6.32	3.7
