# Supplementary material for: Experimental study on soil impermeability based on chemical improvement method
Source: PLoS One. 2025 Oct 21;20(10):e0334100. doi: 10.1371/journal.pone.0334100 (PMC12539693; doi:10.1371/journal.pone.0334100)
Supplement: S1 File — S1 Figure. The original data in Fig 3. S2 Table. The original data in Fig 5. S3 Table. The original data in Fig 6.S4 Table. The original data in Fig 7. S5 Table. The original data in Fig 8. S6 Table. The original data in Fig 9. S7 Table. The original data in Fig 10. S8 Table. The original data in Fig 11. S9 Table. The original data in Fig 12. S10 Table. The original data in Fig 13. S11 Table. The original data in Fig 14. (ZIP) [file pone.0334100.s001.zip › supporting information/S2 Table. The original data in Fig 5.pdf]

S2 Table. The original data in Fig 5

Water Content (%)	Maximum Dry Density (g/cm^3)
10	1.798
12	1.825
14	1.836
16	1.843
18	1.856
20	1.869
22	1.871
24	1.844
26	1.829
