# Supplementary material for: Experimental study on soil impermeability based on chemical improvement method
Source: PLoS One. 2025 Oct 21;20(10):e0334100. doi: 10.1371/journal.pone.0334100 (PMC12539693; doi:10.1371/journal.pone.0334100)
Supplement: S1 File — S1 Figure. The original data in Fig 3. S2 Table. The original data in Fig 5. S3 Table. The original data in Fig 6.S4 Table. The original data in Fig 7. S5 Table. The original data in Fig 8. S6 Table. The original data in Fig 9. S7 Table. The original data in Fig 10. S8 Table. The original data in Fig 11. S9 Table. The original data in Fig 12. S10 Table. The original data in Fig 13. S11 Table. The original data in Fig 14. (ZIP) [file pone.0334100.s001.zip › supporting information/S3 Table. The original data in Fig 6.pdf]

S3 Table. The original data in Fig 6

Erosion Time (min)	Cumulative Erosion Mass (g)			
	SL-C-1	SL-C-2	SL-C-3	SL-C-4
2	52	55	50.25698	51.97577
3	148.0861	98.95619	62.47953	55.41337
4	184.4333	139.0614	72.98328	59.04193
5	240.1657	154.7216	78.13967	65.91712
6	300.7444	215.6433	90.36221	76.42087
7	358.8999	233.0223	114.6163	78.13967
8	472.7879	262.6237	132.1862	86.92462
9	715.1026	292.2252	135.6238	102.5848
10		301.0102	147.8464	107.7412
11		313.2327	151.284	116.5261
12		307.8854	159.878	123.4013
13		307.8854	163.5065	132.1862
14		311.5139	173.8193	140.7802
15		316.6703	179.1667	151.284
16		325.4553	177.4479	156.4404
17		334.0493	182.6043	165.2253
18		330.6117	182.6043	158.1592
19		334.0493	180.8855	158.1592
20		330.6117	180.8855	161.7877
21		330.6117	186.0418	156.4404
22		334.0493	186.0418	159.878
