# Supplementary material for: Experimental study on soil impermeability based on chemical improvement method
Source: PLoS One. 2025 Oct 21;20(10):e0334100. doi: 10.1371/journal.pone.0334100 (PMC12539693; doi:10.1371/journal.pone.0334100)
Supplement: S1 File — S1 Figure. The original data in Fig 3. S2 Table. The original data in Fig 5. S3 Table. The original data in Fig 6.S4 Table. The original data in Fig 7. S5 Table. The original data in Fig 8. S6 Table. The original data in Fig 9. S7 Table. The original data in Fig 10. S8 Table. The original data in Fig 11. S9 Table. The original data in Fig 12. S10 Table. The original data in Fig 13. S11 Table. The original data in Fig 14. (ZIP) [file pone.0334100.s001.zip › supporting information/S4 Table. The original data in Fig 7.pdf]

S4 Table. The original data in Fig 7

Erosion Time (min)	Cumulative Erosion Mass (g)			
	SL-T-1	SL-T-2	SL-T-3	SL-T-4
1	1.13419	1.68591	2	2.19519
2	17.38873	14.96965	10.72564	5.92991
3	28.55046	19.76537	17.38873	9.66464
4	33.09155	29.35683	20.31709	15.52137
5	44.25329	34.40719	28.55046	18.70437
6	72.73056	43.44692	33.34619	27.7441
7	89.53683	62.62983	45.05965	33.09155
8	95.64819	74.89501	52.5291	38.39656
9	105.7489	82.87374	59.19219	39.75464
10	112.9213	87.37238	64.49719	41.87664
11	118.2687	90.04611	77.82337	45.61137
12	123.5738	93.52619	79.94537	43.99865
13	135.0326	95.64819	81.81274	45.61137
14	138.7673	97.51556	88.73046	46.67237
15	139.8283	99.63756	90.85247	42.38592
16	144.0723	106.8099	89.79147	40.00928
17	144.624	107.8709	92.16811	44.25329
18	144.624	109.7383	91.65883	43.44692
19	145.4304	113.2184	93.52619	42.38592
20	147.807	114.7887	92.42275	43.44692
21	148.6134	111.8603	94.84183	42.683
22	151.7964	114.0247	90.59783	42.93764
23	152.6028	114.7887	91.91347	39.45756
24	152.8574	113.2184	93.78083	40.81564
25	154.4701	112.1574	92.97447	41.622
26	153.9184	112.6667	94.03547	--
27	153.9184	114.7887	--	--
