# Supplementary material for: Experimental study on soil impermeability based on chemical improvement method
Source: PLoS One. 2025 Oct 21;20(10):e0334100. doi: 10.1371/journal.pone.0334100 (PMC12539693; doi:10.1371/journal.pone.0334100)
Supplement: S1 File — S1 Figure. The original data in Fig 3. S2 Table. The original data in Fig 5. S3 Table. The original data in Fig 6.S4 Table. The original data in Fig 7. S5 Table. The original data in Fig 8. S6 Table. The original data in Fig 9. S7 Table. The original data in Fig 10. S8 Table. The original data in Fig 11. S9 Table. The original data in Fig 12. S10 Table. The original data in Fig 13. S11 Table. The original data in Fig 14. (ZIP) [file pone.0334100.s001.zip › supporting information/S5 Table. The original data in Fig 8.pdf]

S5 Table. The original data in Fig 8

Erosion Time (min)	Cumulative Erosion Mass (g)			
	MG-C-1	MG-C-2	MG-C-3	MG-C-4
1		6.55822	6.55822	6.55822
2	32	11.55974	11.55974	10.40554
3	148.0861	36.75974	16.56127	11.55974
4	184.4333	65.6147	24.25593	15.40707
5	240.1657	88.31394	45.6086	17.90783
6	300.7444	98.317	51.76432	19.2544
7	358.8999	118.3231	63.11394	25.41012
8	472.7879	114.6681	73.117	27.91089
9	715.1026	133.5201	89.46814	35.41318
10		137.175	91.9689	50.61012
11		149.8712	108.3201	63.11394
12		166.03	108.3201	76.96432
13		179.8804	113.3216	83.31242
14		179.8804	117.1689	88.31394
15		183.7277	122.1704	83.31242
16		184.8819	127.172	84.46661
17		189.8834	131.0193	85.81318
18		201.233	134.6743	79.46509
19		205.0804	132.1735	84.46661
20		213.9292	132.1735	83.31242
21		213.9292	132.1735	86.96738
22		218.9307	132.1735	88.31394
23		215.0834	128.5185	84.46661
24		213.9292	133.5201	85.81318
