# Supplementary material for: Experimental study on soil impermeability based on chemical improvement method
Source: PLoS One. 2025 Oct 21;20(10):e0334100. doi: 10.1371/journal.pone.0334100 (PMC12539693; doi:10.1371/journal.pone.0334100)
Supplement: S1 File — S1 Figure. The original data in Fig 3. S2 Table. The original data in Fig 5. S3 Table. The original data in Fig 6.S4 Table. The original data in Fig 7. S5 Table. The original data in Fig 8. S6 Table. The original data in Fig 9. S7 Table. The original data in Fig 10. S8 Table. The original data in Fig 11. S9 Table. The original data in Fig 12. S10 Table. The original data in Fig 13. S11 Table. The original data in Fig 14. (ZIP) [file pone.0334100.s001.zip › supporting information/S6 Table. The original data in Fig 9.pdf]

S6 Table. The original data in Fig 9

Erosion Time (min)	Cumulative Erosion Mass (g)			
	MG-T-1	MG-T-2	MG-T-3	MG-T-4
1	3.38405	1.59887	1.78878	1.18106
2	10.79068	7.60013	4.5995	4.78941
3	17.77949	14.58895	12.57586	7.97996
4	32.36484	25.14813	19.98249	14.17114
5	43.75965	38.55602	30.35176	21.95759
6	52.11584	46.15256	35.36548	29.74404
7	62.90293	52.34374	45.92466	34.56784
8	74.90545	64.53618	54.12892	35.36548
9	89.30089	74.90545	63.13082	35.55539
10	93.47899	81.70436	68.52436	33.96012
11	107.4566	88.50326	71.525	35.36548
12	110.0774	92.07363	74.52563	33.96012
13	113.0781	94.69443	77.29836	33.7702
14	118.0538	97.46717	76.31081	34.56784
15	122.8396	99.06244	75.893	35.13758
16	126.448	98.26481	76.91854	31.56721
17	131.8415	96.66953	75.32326	31.3773
18	133.019	94.69443	75.32326	33.54231
19	133.2469	97.08734	76.50073	32.36484
20	132.0314	96.28971	74.52563	34.75775
21	134.2344	93.8968	74.90545	33.35239
22	132.2213	95.8719	75.51318	35.13758
23	130.0563	94.69443	73.31018	--
24	131.6516	95.26417	72.51254	--
25	133.6267	97.27726	75.893	--
