# Supplementary material for: Experimental study on soil impermeability based on chemical improvement method
Source: PLoS One. 2025 Oct 21;20(10):e0334100. doi: 10.1371/journal.pone.0334100 (PMC12539693; doi:10.1371/journal.pone.0334100)
Supplement: S1 File — S1 Figure. The original data in Fig 3. S2 Table. The original data in Fig 5. S3 Table. The original data in Fig 6.S4 Table. The original data in Fig 7. S5 Table. The original data in Fig 8. S6 Table. The original data in Fig 9. S7 Table. The original data in Fig 10. S8 Table. The original data in Fig 11. S9 Table. The original data in Fig 12. S10 Table. The original data in Fig 13. S11 Table. The original data in Fig 14. (ZIP) [file pone.0334100.s001.zip › supporting information/S7 Table. The original data in Fig 10.pdf]

S7 Table. The original data in Fig 10

Erosion Time (min)	Cumulative Erosion Mass (g)			
	JN-C-1	JN-C-2	JN-C-3	JN-C-4
1		3.1688	5.29381	3.1688
2	32	5.29381	14.95293	4.32789
3	148.0861	17.07794	20.36204	11.86201
4	184.4333	27.70297	27.70297	17.07794
5	240.1657	41.61211	34.27118	23.45296
6	300.7444	56.68035	39.48711	26.73706
7	358.8999	64.02128	45.86213	25.57797
8	472.7879	67.30538	55.52125	26.73706
9	715.1026	76.96451	59.77127	27.70297
10		81.21452	67.30538	28.86207
11		82.18043	67.30538	35.23709
12		89.71455	69.43039	33.11208
13		107.8737	68.27129	36.39619
14		114.2487	68.27129	32.14617
15		122.7488	70.3963	32.14617
16		130.2829	71.5554	28.86207
17		122.7488	73.6804	26.73706
18		128.1579	70.3963	27.70297
19		131.2488	72.71449	30.02116
20		138.7829	72.71449	26.73706
21		133.3738	69.43039	28.86207
22		133.3738	70.3963	33.11208
23		139.942	71.5554	30.98708
24		139.942	70.3963	30.02116
