# Supplementary material for: Experimental study on soil impermeability based on chemical improvement method
Source: PLoS One. 2025 Oct 21;20(10):e0334100. doi: 10.1371/journal.pone.0334100 (PMC12539693; doi:10.1371/journal.pone.0334100)
Supplement: S1 File — S1 Figure. The original data in Fig 3. S2 Table. The original data in Fig 5. S3 Table. The original data in Fig 6.S4 Table. The original data in Fig 7. S5 Table. The original data in Fig 8. S6 Table. The original data in Fig 9. S7 Table. The original data in Fig 10. S8 Table. The original data in Fig 11. S9 Table. The original data in Fig 12. S10 Table. The original data in Fig 13. S11 Table. The original data in Fig 14. (ZIP) [file pone.0334100.s001.zip › supporting information/S8 Table. The original data in Fig 11.pdf]

S8 Table. The original data in Fig 11

Erosion Time (min)	Cumulative Erosion Mass (g)			
	JN-T-1	JN-T-2	JN-T-3	JN-T-4
1	4.08131	3.71151	2.62386	2.99366
2	16.21947	12.84776	8.99748	7.93159
3	22.2233	14.89254	13.08704	10.80298
4	28.4664	19.69995	16.08896	14.78378
5	36.40624	25.83429	22.83238	16.08896
6	43.12792	32.92577	27.40051	15.74091
7	49.02297	38.82082	27.26999	15.61039
8	57.91995	41.58345	26.18234	15.74091
9	62.85787	42.88863	27.16122	15.26234
10	68.8617	41.9315	25.35573	16.45876
11	70.90648	41.82274	25.35573	17.30712
12	72.58146	41.10489	26.68266	18.02497
13	72.23341	40.01724	26.42162	18.9821
14	71.62433	43.97628	27.40051	17.30712
15	73.19054	43.62823	28.35764	18.26425
16	73.53859	44.34608	29.55405	17.30712
17	75.82265	43.25843	27.16122	16.32824
18	74.75676	42.3013	28.22712	15.50162
19	76.5405	41.34417	26.42162	15.26234
20	74.62624	43.38895	27.74856	15.50162
21	73.06002	41.21365	28.35764	15.26234
22	72.82074	42.17078	26.42162	17.30712
23	75.23532	43.01915	26.18234	15.61039
24	74.14767	41.34417	27.26999	15.37111
25	72.82074	43.86752	25.59501	15.02306
