# Supplementary material for: Experimental study on soil impermeability based on chemical improvement method
Source: PLoS One. 2025 Oct 21;20(10):e0334100. doi: 10.1371/journal.pone.0334100 (PMC12539693; doi:10.1371/journal.pone.0334100)
Supplement: S1 File — S1 Figure. The original data in Fig 3. S2 Table. The original data in Fig 5. S3 Table. The original data in Fig 6.S4 Table. The original data in Fig 7. S5 Table. The original data in Fig 8. S6 Table. The original data in Fig 9. S7 Table. The original data in Fig 10. S8 Table. The original data in Fig 11. S9 Table. The original data in Fig 12. S10 Table. The original data in Fig 13. S11 Table. The original data in Fig 14. (ZIP) [file pone.0334100.s001.zip › supporting information/S9 Table. The original data in Fig 12.pdf]

S9 Table. The original data in Fig 12

Erosion Time (min)	Cumulative Erosion Mass (g)		
	SL-T-4	MG-T-4	JN-T-4
1	2.19519	1.18106	2.99366
2	5.92991	4.78941	7.93159
3	9.66464	7.97996	10.80298
4	15.52137	14.17114	14.78378
5	18.70437	21.95759	16.08896
6	27.7441	29.74404	15.74091
7	33.09155	34.56784	15.61039
8	38.39656	35.36548	15.74091
9	39.75464	35.55539	15.26234
10	41.87664	33.96012	16.45876
11	45.61137	35.36548	17.30712
12	43.99865	33.96012	18.02497
13	45.61137	33.7702	18.9821
14	46.67237	34.56784	17.30712
15	42.38592	35.13758	18.26425
16	40.00928	31.56721	17.30712
17	44.25329	31.3773	16.32824
18	43.44692	33.54231	15.50162
19	42.38592	32.36484	15.26234
20	43.44692	34.75775	15.50162
21	42.683	33.35239	15.26234
22	42.93764	35.13758	17.30712
23	39.45756		15.61039
24	40.81564		15.37111
25	41.622		15.02306
